# Nanotechnology-mediated targeting of tumor angiogenesis

**DOI:** 10.1186/2045-824X-3-3

**Published:** 2011-01-31

**Authors:** Deboshri Banerjee, Rania Harfouche, Shiladitya Sengupta

**Affiliations:** 1 BWH-HST Center for Biomedical Engineering, Department of Medicine, Brigham and Women's Hospital, Harvard Medical School, Harvard-MIT Division of Health Science and Technology, Cambridge, MA 02139, USA

## Abstract

Angiogenesis is disregulated in many diseased states, most notably in cancer. An emerging strategy for the development of therapies targeting tumor-associated angiogenesis is to harness the potential of nanotechnology to improve the pharmacology of chemotherapeutics, including anti-angiogenic agents. Nanoparticles confer several advantages over that of free drugs, including their capability to carry high payloads of therapeutic agents, confer increased half-life and reduced toxicity to the drugs, and provide means for selective targeting of the tumor tissue and vasculature. The plethora of nanovectors available, in addition to the various methods available to combine them with anti-angiogenic drugs, allows researchers to fine-tune the pharmacological profile of the drugs *ad infinitum*. Use of nanovectors has also opened up novel avenues for non-invasive imaging of tumor angiogenesis. Herein, we review the types of nanovector and therapeutic/diagnostic agent combinations used in targeting tumor angiogenesis.

## Introduction

Since Judah Folkman emphasized the 'angiogenic switch' hypothesis for tumor progression in 1991, there has been a tremendous surge in targeting angiogenesis for cancer therapeutics [1]. In the past 30 years, many advances have been made in the field, with the elucidation of various angiogenic molecules that could be targeted to halt angiogenesis, and hence, tumor progression. Angiogenesis, the formation of new capillaries from preexisting vessels, is crucial for ensuring normal embryonic vascular development of all vertebrates, as well as regulating physiological processes such as menses and wound healing in adults [2-4]. Deregulation of angiogenesis hence underlies pathologies characterized by vessel overgrowth (e.g. cancer) as well as vessel insufficiency (e.g. cardiovascular disease, CVD) [4].

It is now well-established that without angiogenesis, tumors cannot grow more than 2 mm in diameter [5-7]. Studies in breast cancer patients have showed that angiogenesis positively correlates with the degree of metastasis, tumor recurrence and shorter survival rates, thus demonstrating the value of angiogenesis as a prognostic cancer marker [1,8]. Tumor angiogenesis essentially entails the same sequences of events as physiological angiogenesis, however, the latter proceeds in an uncontrolled and excessive manner giving rise to leaky and tortuous vessels that are in a constant state of inflammation [6,9]. This is mainly due by an upregulation of angiogenic cytokines and growth factors, most notably the vascular endothelial cell growth factor (VEGF) and Angiopoietin (Ang) families, as well as integrins [10-12]. Integrin α_v_β_3 _is the best-characterized heterodimer that is upregulated in most cancer settings, both on the vasculature and on the tumor cells themselves [13,14]. It is hence not surprising that these molecules are often targeted in both experimental and clinical cancer settings.

As such, the first U.S. Food and Drug Administration (FDA) approved anti-angiogenic therapy was the monoclonal antibody Bevacizumab (Avastin), that targets VEGF proteins overexpressed on colorectal cancer cells and their vasculature [15,16]. In spite of the clinical success of Avastin, the majority of other such anti-angiogenic therapeutic agents have yet to pass phase II clinical trials, suggesting a new paradigm is essential to target aberrant angiogenesis.

### Moving away from conventional chemotherapy

#### Targeting aberrant angiogenesis for cancer therapy

Development of anti-angiogenesis therapy is based on either drugs that prevent the formation of new blood vessels supplying to the tumor (e.g. TNP-470, endostatin, angiostatin), or drugs that damage existing blood vessels (e.g. combretastatin) [17]. The underlying mechanisms of action of these anti-angiogenic drugs are either direct, by targeting endothelial receptors, or indirect, by targeting angiogenic cytokines. These mechanisms of action differ from those of conventional chemotherapy in the following ways: (i) selective targeting of the tumor-associated vasculature instead of the tumor cells themselves; (ii) increased bioavailability of tumor endothelial cells to systemically-administered anti-angiogenic drugs due to their direct contact with blood circulation, whereas tumor cells residing in the distant tumor tissue are less accessible to conventional chemotherapeutic agents; (iii) whereas conventional chemotherapy uses the principle of maximum tolerated dose (MTD), anti-angiogenic therapy is administered in lower doses at a relatively more frequent schedule (metronomic chemotherapy), leading to significantly less systemic toxicity [18,19].

Despite these advantages of anti-angiogenic therapy over conventional chemotherapeutic methods, it still suffers from certain limitations. For instance, as a result of systemic administration, most angiogenic inhibitors often fail to reach the targeted tumor vessels, thus exhibiting a poor biodistribution and pharmacokinetic profile, with associated side effects and low efficacy. A great advance towards this end has come from harnessing the advantages of nanotechnology to more efficiently target and kill tumor-associated vasculature. These advantages are made possible by several parameters, including the size of these nanoparticles that allows them to intrinsically home in to metastasized tumors through the enhanced permeability and retention effect (EPR), their ability to evade the immune system and improve the drug's half-life significantly thus lowering its effective dose 50 (ED_50_), and allow for potent selective targeting due to their high surface density [15,18,20,21]. For these reasons, nanotherapeutics are emerging as the new paradigm for anti-angiogenesis research.

#### Nanoparticle-mediated anti-angiogenesis therapy for cancer

Nanotechnology in cancer therapy includes an arsenal of nano-sized materials, generally ranging in dimensions from 1 nm to a few hundred nanometers in at least one dimension [22]. These nanoparticles are designed to carry therapeutic drugs and imaging agents, which are loaded on or within the nanocarriers by chemical conjugation or simply by encapsulation. Nanoparticle based chemotherapeutic agents are designed such that they can passively or actively target cancer cells.

The leaky vasculature associated with tumors contributes towards the phenomenon of passive targeting by nanoparticles. The tumor vessels have increased permeability due to aberrant angiogenesis, thus allowing nanoparticles with diameters less than 200 nm to passively extravasate into the tumor sites through the EPR effect. These nanoparticles are subsequently cleared by the liver [15,23]. Although many factors, including surface area and chemical modifications, can affect the nanoparticle biodistribution, size remains the limiting factor in achieving passive targeting to tumor sites. As such, nanoparticles with sizes less than 10 nm are cleared by the kidney, whereas those larger than 200 nm often accumulate in the extracellular space, and fail to reach the cancer environment *(20)*. Furthermore, poor lymphatic drainage mechanisms in tumors allow the nanoparticles to be retained in the vicinity of the tumor cells and allow them to release their cargo in a sustained manner [15]. For example, polymer-conjugated angiogenesis inhibitor TNP-470 (caplostatin) was found to accumulate selectively in the tumor vessels by the EPR effect and inhibit hyperpermeability of tumor blood vessels [24,25]. In studies published from our laboratory, we have shown that nanoparticle-conjugated chemotherapeutic agents such as doxorubicin [26,27] and angiogenic small molecule inhibitors [28] can preferentially home into tumors by the EPR effect, resulting in selective vascular shutdown and inhibition of tumor growth.

It should be noted that EPR alone is not always sufficient in targeting the tumor sites and hence is often used in conjunction with active targeting. This combination ensures that nanoparticles are retained in the tumor tissues following their extravasation from leaky vessels. Active targeting of tumor tissues is achieved by chemically arraying ligands on the surface of nanoparticles that can recognize and selectively bind to receptors specifically expressed on tumor cells and vessels. The high surface area to volume ratio of the nanoparticles leads to high local density of ligands for targeting. Nanoparticle-mediated active targeting of the tumor vasculature in anti-angiogenic therapy has been achieved by targeting the VEGF receptors (VEGFRs), α_ν_β_3 _integrins, and other angiogenic factors, as discussed briefly in the following section and in more details in each nanovector category later in this review.

#### Targeting tumor neovasculature

The most prominent modification of nanovectors entails covalently conjugating 'tags' at their surface, in order to increase their targeting potential towards tumorogenic cancer cells and/or their associated vasculature. The main 'tag' used thus far for chemotherapy involves proteins that target the integrin family. As previously mentioned, integrins are key players in the angiogenesis process, and moreover, their upregulation is known to promote survival, growth, and invasion of both tumor and endothelial cells [12]. Integrin α_v_β_3 _has been the most widely used as a targeting moiety on nanovectors due to its pleitropic upregulation in a variety of tumor settings [29-32], some of which have been successfully translated into several clinical trials [12,33]. However, important lacunae remain in the field, mainly owing to the inefficiency of integrin targeting in the long-run.

Nanotechnology-based approaches could remedy this limitation, due to their prolonged half-life and increased targeting efficiency. For instance, perfluorocarbon nanoparticles conjugated to various contrasting agents (Gadolinium, Gd or Fluorine isotope 19, ^19^F) have successfully been linked to an α_v_β_3 _integrin antibody and then visualized by magnetic resonance imaging (MRI) in rabbit and mouse models of tumor angiogenesis [29,32]. These studies open the door for non-invasive detection of various types of cancers in clinical settings, as well as for other diseases characterized by aberrant vasculature, such as atherosclerosis and other CVD [31]. In an analogous manner, another approach to target integrin overexpression consists of using a synthetic peptide containing the recognition site for integrins, namely an Arginine-Glycine-Aspartic acid (RGD) sequence [30]. Recent studies are further optimizing integrin targeting by engineering novel peptide moieties which bind with better affinity to integrins than current RGD tags [34,35].

Another characteristic of tumor-associated vasculature is inflammation, resulting in upregulation of various markers known to promote endothelial-tumor cell interactions and metastasis, such as endothelial-cell selectin (E-selectin) [36]. Although E-selectin-based nanotherapeutics have been used less extensively than integrin-targeting nanoparticles, they do provide an additional means to target activated endothelium, and might hence provide an attractive tag to be used in conjunction with integrin targeting [37].

It becomes apparent that targeting-based approaches for tumor therapies are only as good as the selectivity and specificity of the targeting moiety used. This, in turn, implies that using disease-selective markers is crucial in order to obtain maximum selectivity without deleterious side-effects. Since targeted nanotechnology is often coupled with a chemotherapeutic agent entrapped in the nanoparticle, proper targeting to the diseased tissue is crucial to minimize systemic side-effects [38]. As most diseased states are usually characterized by several markers, an attractive direction would be to combine several tags on one nanovector, so long as these do not interact with each other.

### Engineering anti-angiogenic nanoparticles to suit our needs: Playing with nanovector backbone and drug coupling for therapeutic and imaging purposes

Since nanoparticles were first proposed by Marty JJ. *et al*. in 1978 as novel drug-delivery systems [39], their use as anti-cancer agents exploded during the 1980 s. However, only more recently (1995) have they been used to target angiogenesis [40]. Several nanovectors have been reported thus far in mediating anti-angiogenesis therapy and imaging of the tumor vasculature. These include an arsenal of synthetic and natural nanoparticles such as polymeric conjugates and polymeric nanoparticles; liposomes and micelles; synthetic organic nanoparticles such as dendrimers; carbon-based nanostructures such as carbon nanotubes and polyhydroxylated fullerenes; inorganic nanoparticles of gold, silver and iron-oxide; quantum dots; viral capsids and ferritin. The plethora of nanovectors allows researchers to fine-tune the properties of the drugs depending on their target. Further fine-tuning is also possible depending on the method of drug-nanovector coupling, thus offering the potential to engineer revolutionary therapeutics in the field of angiogenesis. Herein, we review the different types of nanovectors that have been studied to formulate anti-angiogenic agents for imaging and therapeutic purposes, their main modifications, as well as their advantages and limitations.

#### Polymeric nanoconjugates

A diverse family of polymers has been studied for the engineering of nanoparticle-based drug delivery agents since one of the earliest reports in 1979 describing their use in cancer therapy [41]. Polymers chemically conjugated to drugs are regarded as new chemical entities owing to their distinctive pharmacokinetic profile as compared to the parent drugs. Polymeric nanoparticles can also be designed to encapsulate drugs without any chemical modification. Encapsulated drugs can be control-released from the polymer matrix by diffusion or through surface or bulk erosion, while release of conjugated drugs requires cleavage of the covalent bonds under biological conditions. Some key examples of polymer-based nanoconjugates for anti-angiogenesis therapy have been prepared from structures, such as N-(2-hydroxypropyl)methacrylamide (HPMA), poly(lactic co-glycolic acid) (PLGA), polysaccharides (e.g. chitosan) and dendrimers, to name a few.

##### N-(2-hydroxypropyl)methacrylamide (HPMA) copolymers

HPMA copolymers are hydrophilic substances that have been extensively studied for their anti-angiogenesis potential. An HPMA copolymer conjugated to the angiogenesis inhibitor TNP-470 (Caplostatin), was the first polymer-conjugated angiogenesis inhibitor reported [25]. It was found to selectively accumulate in the tumor microvasculature, resulting in decreased tumor growth rate in human melanoma and lung carcinoma mice models. Interestingly, such formulation of TNP-470 prevented it from crossing the blood-brain barrier, thus overcoming the neurotoxicity often associated with chemotherapeutic drugs. The mechanisms of actions underlying Caplostatin's chemotherapeutic effects included inhibition of various angiogenic signaling pathways such as: VEGF receptor-2 (VEGFR-2), mitogen-activated protein kinase (MAPK) and RhoA [24]. HPMA copolymers have also been used to design novel bone-targeted anti-angiogenic therapeutic agents [42,43]. In these studies, the bone-targeting aminobisphosphonate drug alendronate (Fosamax) was co-conjugated to the polymer backbone along with a chemotherapeutic drug (e.g., paclitaxel, TNP-470), thus inhibiting bone metastasis. Here, passive targeting was achieved by extravasation of the nanoconjugates from the leaky tumor vessels via the EPR effect, while active targeting to the calcified tissues was achieved by alendronate's high affinity to the bone mineral. These studies have tremendous clinical implications, as bone metastasis is associated with a plethora of cancers, and its presence correlates with the terminal stage of cancers.

Specific peptide sequences have been conjugated to HPMA copolymers for active targeting of the α_ν_β_3 _integrin in tumor-associated vasculature. Radionuclide-labeled, cyclized RGD peptide-tagged HPMA copolymer-based nanoconjugates have been designed that provide the potential for targeted delivery of radionuclides and drugs to solid tumors for diagnostic and therapeutic applications [44,45]. The conjugates exhibited increased tumor retention times and rapid clearance from normal tissues, thus reducing systemic toxicity associated with standard therapeutics. These studies clearly demonstrated the significance of α_ν_β_3 _targeting using RGD-bearing conjugates, as this could provide a promising strategy for selective delivery of angiogenesis inhibitors and imaging agents to tumor vasculature and tumor sites expressing the α_ν_β_3 _integrin.

##### Poly(lactic co-glycolic acid) (PLGA) copolymers

**Figure 1:**
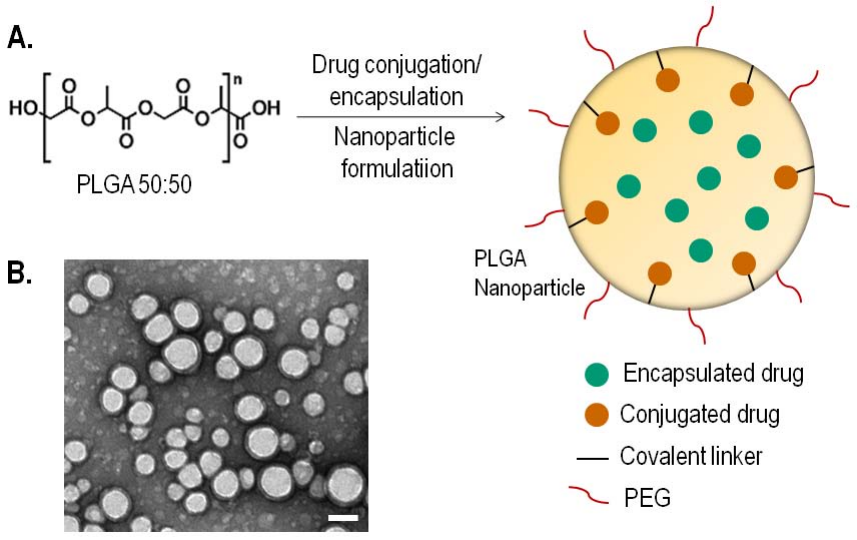
**PLGA nanoparticles:** (A) Chemical conjugation or simple encapsulation of chemotherapeutic agents in PEG-modified PLGA nanoparticles. (B) Transmission electron microscopy image of PLGA nanoparticles. Scale bar: 100 nm.

PLGA copolymers have been extensively used in the field of cancer research, owing to their biodegradability and biocompatibility, resulting in their FDA approval. PLGA is synthesized by the co-polymerization of two different monomers, lactic acid and glycolic acid, and can be further modified chemically for conjugation or simple encapsulation of drugs in a nanoparticle formulation, as shown in Figure 1. In a study targeting the MAPK signaling pathway, Basu and Harfouche *et al*. have reported the use of PLGA copolymer for chemically conjugating PD98059, a selective MAPK inhibitor [46]. The resulting nanoparticles selectively resulted in melanoma regression in a mouse model. In a consecutive study, Harfouche and Basu *et al*. encapsulated an inhibitor of the phosphatidylinositol-3-kinase (PI3K) pathway in a PLGA copolymer, and used novel zebrafish melanoma and breast adenocarcinoma tumor xenograft models to demonstrate its anti-angiogenic effect [28].

PLGA-based nanoparticles have also been used to engineer complex nanosystems. In a novel strategy reported by Sengupta *et al*., temporal targeting of tumor cells and the tumor vasculature was achieved using a nanoscale delivery system, described as a 'nanocell', that comprised of a core PLGA nanoparticle encapsulated within a polyethylene glycol (PEG)-linked lipid envelop [26]. PEGylation of a molecule renders the latter non-toxic and non-immunogenic, and is an FDA approved method [47]. In this nanostructure, the chemotherapeutic drug doxorubicin was covalently attached to the inner PLGA core, and the anti-angiogenic agent combretastatin was trapped within the outside lipid envelope. After disruption of the outer envelope inside a tumor, release of combretastatin led to vascular collapse and intra-tumoral trapping of the nanoparticles, which subsequently released the chemotherapeutic drug in response to local hypoxia, resulting in significant regression of various tumors including melanoma. The nanocell is an example of nanoparticles being engineered based on understanding of the disease, and can be fine-tuned to optimize chemotherapy, thus shifting the paradigm from conventional anti-angiogenic treatments.

**Figure 2:**
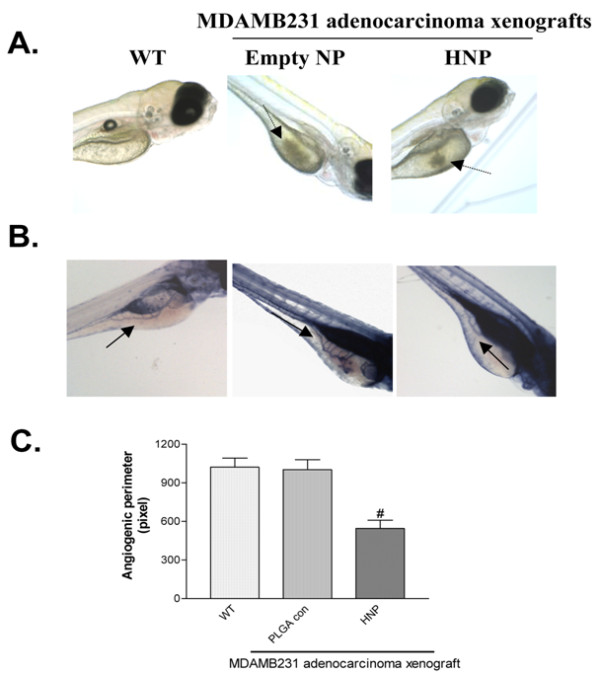
**Effects of LY-encapsulated PLGA nanoparticles *in vivo *using Casper Zebrafish-breast adenocarcinoma xenograft assay**. (A) Real-time imaging. Broken arrows show cancer cells. (B) Alkaline phosphatase vessel staining. Full arrows show subintestinal vessels (SIV). (C) Quantification of SIV using morphometric analyses developed in our laboratory. HNP = hybrid nanoparticles. P^# ^≤ 0.05 versus wild-type and empty nanoparticle controls. (In collaboration with Dr. Richard M. White and Dr. Leonard I. Zon, Children's Hospital Boston).

In our ongoing studies using a Zebrafish mutant, named Casper, which allows the visualization of cancer cells in real-time even when they are unlabeled [48], we demonstrated that hybrid nanoparticles of PLGA carrying an inhibitor of PI3K not only inhibited vascularization, as shown by reduced subintestinal vessel (SIV) density, but also inhibited both human melanoma and breast adenocarcinoma tumor cell growth (Figure 2).

PLGA nanoparticles have not only been used as delivery agents of synthetic drugs, but have also recently been described for delivering natural products thought to have anti-cancer effects. Curcumin (yellow pigment in the spice turmeric)-loaded PLGA nanoparticles were reported to successfully suppress tumor necrosis factor (TNF)-regulated expression of VEGF, culminating in reduced tumor metastasis [49]. This approach is promising for using natural products for chemotherapeutics, as it eliminates many side-effects observed with synthetic drugs.

##### Polysaccharides and Dendrimers

Polysaccharides (e.g. chitosan) and dendrimers can also be considered as polymeric systems, and have been studied as carriers of anti-angiogenic agents for therapeutic applications. Chitosan is a commercially available cationic linear polysaccharide, which has found a variety of applications in pharmaceuticals and biomedicine. Dendrimers, on the other hand, possess a branched structure that provides it with certain unique properties like ease of chemical conjugation, biocompatibility, high water-solubility and easy renal clearance due to their small size [15,50]. Different dendrimeric systems are now under investigation for novel cancer treatments [50]. In a recent study, chitosan nanoparticles have shown significant inhibition of tumor growth and induction of tumor necrosis in a mouse hepatocellular carcinoma xenograft model [51]. The anti-tumor activity of these nanoparticles was found to be related with their anti-angiogenic activity, which was linked to significant reduction in the levels of VEGFR-2 expression and subsequent blockage of VEGF-induced endothelial cell activation. In using dendrimers as nanovectors, Backer *et al*. have described the construction of VEGF_121_-containing, boronated polyamidoamine dendrimer that can be used to target VEGF receptors on tumor neovasculature [52]. Near-IR fluorescent imaging of mouse breast carcinoma revealed selective accumulation of these dendrimers in the periphery of growing tumors, where tumor neovascularization was most prominent. These studies demonstrate the potential of designing chitosan and dendrimer-based nanoparticles for both chemotherapeutic and imaging purposes.

#### Lipid-based nanoparticles

Lipid-based nanocarriers, such as liposomes and micelles, possess attractive biological properties, including biocompatibility, biodegradability, and the ability to entrap both hydrophobic and hydrophilic drugs [15]. Liposomes are FDA-approved spherical structures consisting of phospholipid bilayers with an enclosed aqueous phase that can carry a range of chemotherapeutic drugs. Micelles, on the other hand, consist of lipid monolayers with a hydrophilic shell enclosing a hydrophobic core in a spherical structure. Although liposomal nanosystems have been widely reported for anti-angiogenic therapy, reports on using micelles are relatively fewer. Benny *et al*. reported that conjugation of the angiogenesis inhibitor TNP-470 to monomethoxy-polyethyleneglycol-polylactic acid copolymer resulted in the formation of nanopolymeric micelles, named Lodamin [53]. On oral administration, the conjugate was found to accumulate selectively in tumors, inhibiting tumor growth, angiogenesis and proliferation, without causing any neurological impairment in tumor-bearing mice. In another study based on targeting the ανβ3 integrin, Nasongkla *et al*. designed a cyclic RGD pentapeptide conjugated, doxorubicin-loaded poly(*ε*-caprolactone)-polyethyleneglycol (PCL-PEG) nanopolymeric micelles [54]. The micelles showed high efficiency in targeting SLK tumor endothelial cells derived from human Kaposi's sarcoma *in vitro*. Since most chemotherapeutics are delivered in highly invasive manners, such as by systemic injection, this method of targeted delivery has the crucial capacity of immensely reducing discomfort and cytotoxicity in the patient.

Adding to the library of peptide-tags for targeting is a pentapeptide sequence consisting of the amino acids Alanine-Proline-Arginine-Proline-Glycine (APRPG), that has been isolated from a phage-displayed peptide library and has been found to specifically bind to tumor angiogenic vasculature [55]. APRPG-conjugated liposomal nanosystems containing chemotherapeutic drugs have been studied for their vasculature-targeting anti-angiogenic effects [56,57]. These studies demonstrated the benefits of using the APRPG-motif for active targeting of drug carriers to angiogenic site in a novel tumor-treatment modality, namely anti-neovascular therapy [58].

Due to their ease of synthesis, liposomal systems have been used to target a plethora of angiogenic factors, including VEGFRs, ανβ3 integrins and matrix metalloproteinases (MMPs). Li *et al*. investigated the potential anti-angiogenic efficacy of two polymerized liposomal nanoparticles radiolabeled with Yttrium isotope 90 (^90^Y), one conjugated to a small molecule integrin antagonist targeting the ανβ3 integrin and the other loaded with a monoclonal antibody against murine VEGFR-2 receptor Flk-1 [59]. Membrane type-1 matrix metalloproteinase (MT1-MMP), expressed on angiogenic endothelium cells and tumor cells, also plays an important role in angiogenesis. Hatakeyama *et al*. designed Fab' fragments of anti-human MT1-MMP monoclonal antibody conjugated to PEG-modified doxorubicin-encapsulating liposomes that showed significant decrease in tumor volume *in vivo *as compared to the non-targeted liposomes [60].

#### Carbon nanovectors

Carbon-based nanostructures, such as carbon nanofibers, nanotubes and fullerenes, have received considerable attention for cancer research in the past. This is due to several advantages, including: (1) high mechanical strength and surface area, (2) numerous sites for chemical or physical conjugation, (3) light weight properties, and (4) ease of scalability and manufacturing [61]. However, issues related to their biocompatibility, renal clearance and toxicology have since limited their use in biomedical applications. In addition, although an increasing number of studies have been reported on the use of carbon nanovectors in cancer research, their use in targeting angiogenesis remains limited.

**Figure 3:**
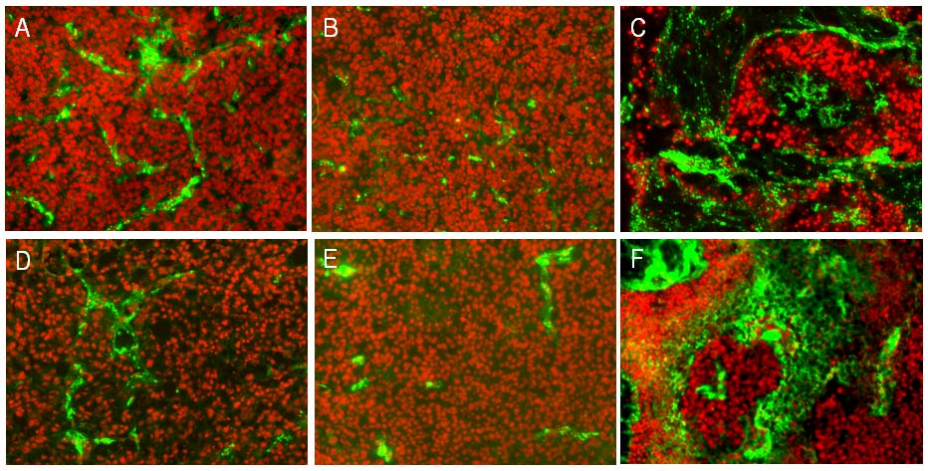
**Effects of doxorubicin (Dox)-conjugates of fullerenols and carbon nanotubes (CNT) on B16-F10 mediated angiogenesis in mouse xenograft model**. Angiogenesis was assessed by immunodetection of the Von Willebrand Factor (vWF) endothelial cell marker (green) and propidium iodide counterstain (red) on treatments with: (A) control; (B) fullerenol; (C) CNT; (D) Dox alone; (E) fullerenol-Dox conjugate; and (F) CNT-Dox conjugate.

Murugesan *et al*. have reported the efficacy of multiwalled carbon nanotubes, C_60 _fullerenes and graphite in inhibiting VEGF- and bFGF-induced angiogenesis in chick chorioallantoic membrane (CAM) [61]. These carbon materials did not have any significant effect on basal angiogenesis in the absence of added growth factors, indicating potential in tumor environment where angiogenic factors are known to be upregulated. In an interesting study, Chaudhuri *et al*. reported that the shape of carbon-based nanostructures markedly affects the chemotherapeutic potential of doxorubicin [27]. The authors showed differential effects between doxorubicin (Dox) conjugated to single-walled carbon nanotubes (CNT) versus spherical fullerenols (Full) on angiogenesis (Figure 3). Both empty and Dox-conjugated fullerenols exerted anti-angiogenic effects in zebrafish and mouse melanoma models. In contrast, empty and Dox-conjugated CNTs exerted a pro-angiogenic effect both *in vitro *and *in vivo*. Mechanistic studies implicated differential activation of ανβ3 integrins and downstream PI3K signaling between CNT and fullerenol in endothelial cells, which implicates the role of nanovector shapes in mediating drug fate, and underlies the importance of choosing the right vector to obtain optimal therapeutic index.

#### Inorganic nanoparticles

Inorganic nanoparticles, such as the ones derived from gold, silver and iron-oxide, possess unique optical, electrical, magnetic, and photothermal properties, which have been harnessed in numerous biomedical applications. Recently, they have been reported for use in anti-angiogenic therapy also.

Mukherjee *et al*. observed that gold nanoparticles bind specifically with heparin-binding growth factors, such as VEGF_165 _and bFGF, resulting in inhibition of endothelial cell proliferation *in vitro*, and VEGF-induced permeability and angiogenesis *in vivo*, but failed to inhibit the activity of non-heparin binding growth factor VEGF_121 _[62]. Gurunathan *et al*. reported on the anti-angiogenic properties of silver nanoparticles, and demonstrated that these agents could inhibit VEGF-induced cell proliferation, migration and formation of new blood microvessels *in vivo *[63]. Furthermore, their results indicated that silver nanoparticles could target the activation of PI3K/Akt signaling pathways, thus leading to the inhibitory effect of angiogenesis.

The paramagnetic properties of iron-oxide nanoparticles have been harnessed for therapeutic and imaging applications [64]. Chen *et al*. have reported that dextran-coated iron-oxide nanoparticles conjugated to radiolabeled (Iodine isotope 131, ^131^I) anti-VEGF monoclonal antibody significantly increased imaging resolution as well as destruction of liver cancer in mice [65]. Maeng *et al*. have developed doxorubicin-loaded, folate-receptor targeted superparamagnetic iron oxide nanoparticles that significantly inhibited tumor growth, yet surprisingly, did not increase systemic cytotoxicity often associated with heavy metals, most likely due to their selective localization in tumors [66].

#### Imaging tumor angiogenesis

Medical imaging has undergone tremendous research and development over the past few decades, with the introduction of techniques such as magnetic resonance imaging (MRI), computed tomography (CT), ultrasonography, nuclear medicine scanning, and optical fluorescence imaging [67,68]. The possibility of non-invasive and accurate monitoring of tumor response has led to growing interest in the use of these techniques in angiogenesis research. Use of nanoparticles offer several advantages in this area of research, including their capability of carrying high payloads of therapeutic and diagnostic agents, improved contrast, and longer circulation times in the body.

MRI has been found to correlate more directly with tumor angiogenesis as compared to other imaging techniques [67]. Drevs et al. have reported the use of the dynamic enhanced MRI technique for studying the effects of PTK787/ZK 222584, a specific VEGF receptor tyrosine kinase inhibitor, on the anatomy and functional properties of tumor vessels [69]. Dextran-associated, superparamagnetic iron oxide nanoparticles (Endorem) were used in this study to detect the partial tumor blood volume in a murine renal cell carcinoma model. Reichardt et al. have also used the MRI technique and superparamagnetic nanoparticles in imaging the anti-angiogenic effects of a small molecule VEGF receptor tyrosine kinase inhibitor in a drug-resistant human adenocarcinoma model [70]. Sipkins et al. have pioneered the use of endothelial ανβ3-targeted, gadolinium ion-containing paramagnetic liposomes for imaging tumor angiogenesis by MRI [71]. Intravenous administration of these nanoparticles provided detailed and enhanced imaging of the ανβ3 expressing tumor vasculature in rabbit carcinomas.

A combination of two or more modalities, either in imaging or in therapeutic applications, or both, can function synergistically to provide complementary informations. A bimodal approach to imaging tumor angiogenesis, using MRI and fluorescence, was reported by Mulder et al [72]. In a following study, the same bimodal liposomal system was used for non-invasive evaluation of the therapeutic efficacy of angiogenesis inhibitors anginex and endostatin [73]. In a novel approach, Kluza et al. have functionalized a bimodal (MRI- and fluorescence-detectable) paramagnetic liposomal nanoparticle with two angiogenesis-specific targeting ligands - an ανβ3-specific RGD peptide sequence and a galectin-1-specific designer peptide anginex [74]. This strategy of dual ligand targeting provided synergistic targeting effects *in vitro *significantly improving the uptake of these nanoparticles as compared to those modified for single ligand targeting.

Apart from MRI and optical imaging, liposomes have also been used to encapsulate contrast agents for CT imaging. For example, Samei et al. have reported the use of a long-circulating liposomal system encapsulating traditional iodinated contrast agent for micro-CT imaging of rats implanted with R3230AC mammary carcinoma [75].

**Figure 4:**
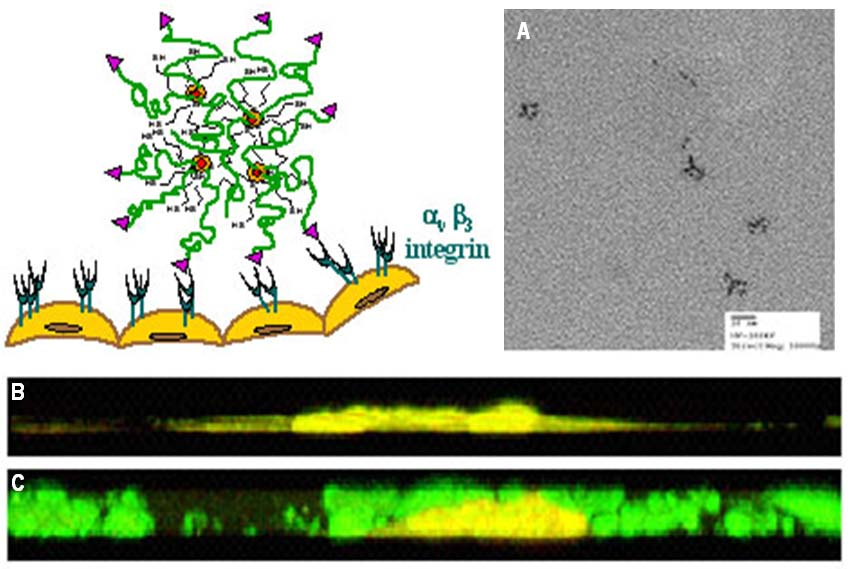
**PEGylated Quantum Dot (QD) nanocrystals conjugated to ανβ3-specific RGD peptide for endothelial cell targeting;** (A) low resolution transmission electron microscopy image showing the nanoconjugates, the arrow indicating one such nanostructure; (B and C) spinning disk confocal microscopy images showing uptake of RGD-targeted QD clusters in endothelial cells treated with cations that are known to cause integrin clustering. Incubation with the RAD-PEG-nanocrystal QD clusters showed no uptake by the cells (B), while RGD-PEG-nanocrystal QD clusters resulted in increased uptake into the cells (C).

Quantum dots (QDs), which are fluorescent nanocrystals made of inorganic semiconductor materials, have gained prominence in biomedical imaging due to their unique photostable and fluorescence properties. We observed that QD complexes, conjugated to ανβ3 integrin-binding cyclic RGD peptide for endothelial cell targeting *in vitro*, showed increased uptake into the cells as compared to QDs that were conjugated to a control RAD (Arginine-Alanine-Aspartic acid) peptide (negative control) (Figure 4).

Paramagnetic QDs have been engineered as a bimodal (MRI and fluorescence) imaging probe. Such QDs functionalized by ανβ3-specific RGD peptide have been used for successful targeting of human endothelial cells *in vitro *[76]. In another study, paramagnetic QDs on conjugation with a cyclic peptide that can target CD13, an aminopeptidase highly overexpressed on angiogenic tumor endothelium, enabled quantitative molecular MRI of tumor angiogenesis *in vivo *[77].

Natural nanoparticles, such as viruses and ferritin, have several advantages over synthetic nanoparticles, including precise dimensions, possible evasion by the immune system, biocompatibility and biodegradability [68]. A number of viral nanoparticles (VNPs) have been developed for targeted delivery and imaging purposes, e.g. cowpea mosaic virus (CPMV) and bacteriophages such as MS2, Qβ, and their modes of cellular uptake have been studied [78]. Banerjee *et al*. have recently reported the mechanism of receptor-mediated endocytosis of transferrin-decorated bacteriophage Qβ [79]. Multivalent display of fluorescent tags on CPMV facilitated intravital visualization of human fibrosarcoma-mediated tumor angiogenesis in a CAM model [80]. In a different study, covalent attachment of a VEGF receptor-1 specific peptide to fluorescently labeled CPMV enabled efficient targeting of the VNPs to VEGFR-1-expressing tumor *in vivo *[81].

Apart from viral nanoparticles, protein cages such as the iron-containing ferritin have also been studied in medical imaging [68]. Crich et al. have reported the MRI visualization of tumor angiogenesis *in vivo *by targeting the neural cell adhesion molecules expressed on tumor endothelium with a highly sensitive gadolinium-containing apoferritin probe [82].

### New generation research in anti-angiogenesis therapy

Apart from the more conventional approaches of arraying small molecule chemotherapeutic drugs or antibodies on different synthetic or natural nanovectors to achieve anti-angiogenic effects, new research reports are emerging that target the molecular mechanism of angiogenesis by using approaches such as gene silencing and others. In the following sections, we will review some of these emerging new strategies.

#### Nanoconjugates for siRNA/gene delivery

An interesting strategy to target tumor vasculature is by systemic delivery of an anti-angiogenic gene using a nanoconjugate system. The soluble fragment of VEGF receptor Flt-1 (sFlt-1) is a potent and selective inhibitor of VEGF. As such, Kim *et al*. showed that the stable expression of sFlt-1 by endothelial cell targeted gene delivery inhibited angiogenesis [83]. For this purpose, the authors designed a PEGylated polyethyleneimine (PEI) nanosystem, consisting of the ανβ3 integrin targeting RGD peptide sequence, PEI-*g*-PEG-RGD, and complexed with the therapeutic gene encoding sFlt-1. The PEI-*g*-PEG-RGD/pCMV-sFlt-1 nanoconjugate system successfully inhibited the proliferation of cultured endothelial cells *in vitro *by blocking the binding of VEGF to the membrane-bound full length Flt-1 receptor. In a further study, the authors showed that this polymeric gene delivery system reduced tumor burden in mice while increasing prognosis [84].

The use of small interfering RNA (siRNA) and short-hairpin RNA (shRNA) have tremendously helped in our understanding of the molecular mechanisms underlying angiogenesis and tumor developments. The high specificity of these small RNA sequences in binding target proteins post-transcriptionally can prove crucial to our efforts in designing new generation of therapeutics [85]. In one such study, Schiffelers *et al*. have reported the engineering of RGD-sequence bearing PEGylated self-assembling polymeric nanoparticles that can be used to deliver siRNAs specifically targeted to inhibit VEGFR-2 expression, thereby inhibiting tumor angiogenesis [86]. Intravenous administration of these nanoparticles into mice bearing neuroblastoma N2A tumors led to selective tumor uptake, siRNA sequence-specific inhibition of VEGFR2 expression in tumor, and inhibition of tumor growth and angiogenesis.

Lipid-based nanosystems have also been reported for targeted gene and siRNA delivery. Protease-activated receptor-1 (PAR-1) siRNA incorporated into neutral liposomes was used to target the thrombin receptor PAR-1, which is involved in adhesion, invasion and angiogenesis [87]. Systemic delivery of these nanoparticles inhibited both melanoma tumor growth and metastasis in mice. This led to significant inhibition of tumor growth and weight, in addition to a concomitant decrease in the expression of various angiogenic factors (VEGF, interleukin-8 and MMP-2), as well as reduced blood vessel density.

Apart from polymeric and lipid-based nanosystems, chitosan nanoparticles loaded with siRNA have also been designed to provide a novel therapeutic tool for selectively knocking down angiogenesis genes. For instance, Pillé *et al*. have reported the use of a chitosan-coated polyisohexylcyanoacrylate nanoparticle for encapsulating anti-RhoA siRNA, resulting in inhibition of tumor growth and angiogenesis in an aggressive breast cancer mouse xenograft model [88].

#### Aptamers

Aptamer-based nanotherapeutics have emerged as a novel strategy due to their recognition, and hence targeting, of an endless list of moieties including oncogenes, viruses, bacteria and inflammatory proteins. Aptamers are short, three-dimensional synthetic RNA or DNA oligonucleotides (15-40 nucleotides long) or peptides (10-20 amino acids long) that bind their target with high affinity and specificity, hence their nickname of 'chemical antibodies' [89-91]. Depending which epitope aptamers bind to, namely an active versus non-active site, determines whether aptamers inhibit function or simply acts as a targeting moiety. These properties make aptamers ideally suited for diagnostics, imaging and targeting of angiogenic-based pathologies. The strengths of aptamers reside in their versatility, non-immunogenicity, low cost, high reproducibility and ease of production, which become immediately apparent when compared with standards antibodies. For instance, oligonucleotide and peptide aptamers can be isolated from an impressively large repertoire of libraries, by processes known as Systematic Evolution of Ligands by Exponential Enrichment (SELEX) and yeast/bacterial expression libraries, respectively [89,91]. These libraries have been key determinants in increasing the efficacy and popularity of aptamer-based nanotechnology, as they allow high-throughput screening and can hence find disease markers. The main lacuna of aptamers are that they are easily degraded by cellular nucleases and proteinases [89]. This lacuna is easily remedied by chemical modifications of the aptamers, making them ideal candidate for novel nanotherapeutics with improved targeting power and therapeutic index [90].

Oligonucleotide aptamers have recently gained much popularity in the field of cancer in general. For instance, they have played a crucial role in increasing targeting and imaging of cancer cell and their associated vasculature by being coupled to metallic nanoparticles such as magnetic, gold and ruby-eye doped nanoparticles, as well as quantum dots [90,92-94]. This coupling of aptamers to metallic nanoparticles creates an exciting new opportunity for tumor detection, as it potentiates the biosensor capability of metallic nanoparticles significantly. Using other type of nanovectors, VEGF-targeting RNA aptamers have been combined with either PLGA microspheres or 1,2-distearoyl-*sn*-glycero-3-phosphatidylcholine (DSPC) and cholesterol nanoliposomes, resulting in potent and selective inhibition of angiogenesis *in vitro *and *in vivo *for prolonged time periods [95,96]. In another interesting study, an Ang-2 RNA aptamer was shown to inhibit adult neovascularization in a rat corneal pocket model of tumor angiogenesis [97]. As the role of Ang-2 in mediating vascularization has long been contested, with contradicting reports demonstrating both pro- and anti-angiogenic roles, this study validates our previous findings that Ang-2 can indeed play a preponderant role in promoting angiogenesis [98].

Oligonucleotide aptamers have preferentially been used, as compared with their peptide counterparts, mainly due to their higher binding affinities and lower costs and ease of synthesis and purification. This has translated into very few reports combining peptide aptamer- and nanoparticle-based technologies, with most studies instead focusing on the advantages of these aptamers for forward and reverse genetic experiments [91]. Hence, there is currently an enormous gap with respect to peptide aptamer-based nanotherapeutics, altough the potential is certainly present, mainly due to the advantage of peptide aptamers in disrupting protein-protein interactions. As such, they have efficiently been used in cancer setting to target and inhibit various tumor markers associated with tumor growth and metastasis [99,100]. Although these latter studies didn't conjugate the aptamers with nanovectors, they open up the doors to novel and exciting possibilities in the nanotechnology field. To the best of our knowledge, peptide aptamers have only been conjugated with nanomaterials in the case of carbon, but for the sole purpose of increasing its solubility [101].

#### Stem cell-based nanotherapeutics

**Figure 5:**
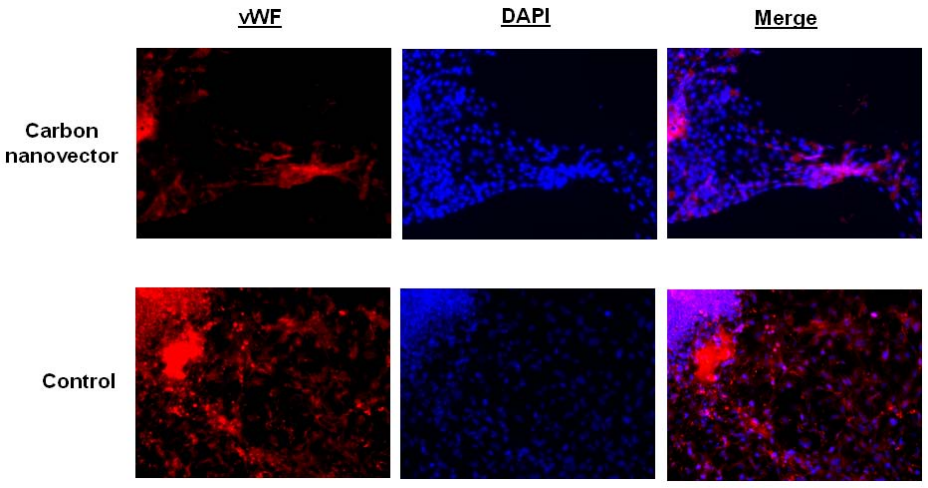
**Carbon nanovectors inhibit stem cell-mediated vascularization**. Immunocytochemistry results show Von Willebrand Factor (vWF) endothelial cell marker (red) and DAPI counterstain (blue).

In a novel approach, embryonic stem cells have been used in conjunction with nanoparticles for either tumor imaging [102] or for pro-angiogenic therapy during CVD-related ischemia [103]. For imaging, stem cells were labeled with ferumoxides-poly-lysine complexes (e.g. iron oxide superparamagnetic nanoparticles) and used to visualize gliomas using MRI. This finding has enormous therapeutic implications, since very few agents are able to bypass the blood brain barrier, often resulting in insurmountable obstacles for the treatment of brain malignancies. Regarding pro-angiogenic stem cell-based therapeutics, cells were transfected with VEGF DNA using biodegradable poly(β-amino esters) nanoparticles, leading to significant vascular regeneration in ischemic tissues [103]. This methodology in itself is quite novel, as standard transfection methods rely on plasmids or electroporation, which results in high cell stress and low efficiency. Although there are very few studies merging nanotechnology with stem cells, this field warrants further investigation due to its immense clinical potential in a variety of angiogenic diseases, especially those characterized by vessel insufficiency. In a novel approach, our laboratory has attempted to merge the use of carbon nanovectors with embryonic stem cells. Our preliminary results show that the shape of carbon nanostructures can modulate stem cell fate, with an example of an inhibitory effect on vascularization, as shown in Figure 5. Our ongoing studies underlie the infinite possibilities of modulating tissue regeneration with, not only scaffold type, but structure as well.

## Concluding Remarks and Future Directions

The tumor neovasculature is an attractive target for anti-angiogenic therapy as well as non-invasive imaging studies. Nanotechnology has emerged as an exciting field in this area of research due to multiple advantages, including the capacity of nanoparticles to carry multiple moities of therapeutic and imaging agents, offer longer circulation time and increase the therapeutic index of chemotherapeutcs, to name a few. Moreover, with the various types of nanovectors available, many of which are FDA-approved, along with the various methods for coupling them to drugs and diagnostic agents, there is an endless opportunity to fine-tune nanotherapeutics depending on the task needed. Clearly, the advent of nanothechnology provides a huge potential for devising increasingly novel anti-angiogenic therapeutics that can eventually be translated from bench to bed-side.

## Competing interests

The authors declare that they have no competing interests.

## Authors' contributions

DB and RH collected the references and compiled the manuscript; DB, RH and SS edited the manuscript. All authors read and approved the final manuscript.
